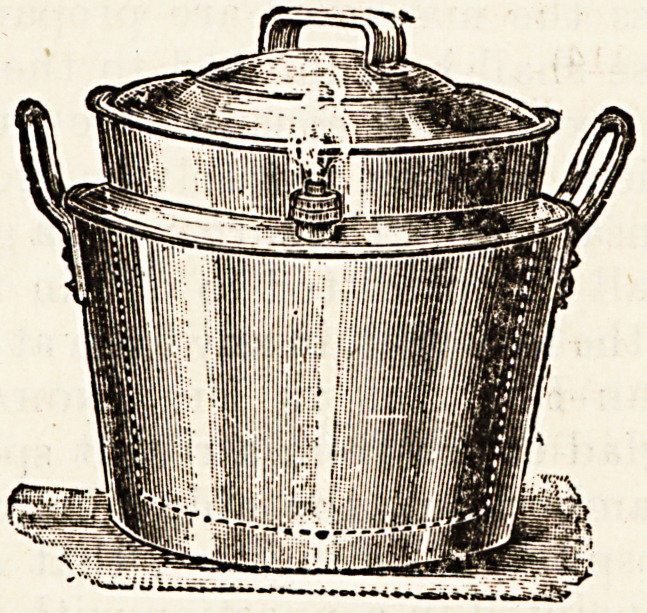# A Useful New Appliance

**Published:** 1907-04-13

**Authors:** 


### A USEFUL NEW APPLIANCE.

### WELBANK'S BOILERETTE.

This excellent saucepan, manufactured by Messrs. Welbank, Duplex Works, near Banbury, maintains it reputation as one of the most perfect cooking utensils that can be found on the market. We have given it a careful, and in many respects a severe trial, and find that the manufacturers by no means overrate its merits when they state that it is a "perfect cooker." Porridge made in the boilerette is a revelation to anyone accustomed only to the product of the ordinary enamelled saucepan; that most difficult of beverages to prepare perfectly, chocolate, can easily be made in the boilerette. Soups and vegetables, fish and meats, are all suitable for the boilerette, and we have found it no less useful for cookery of a lighter kind—egg dishes and the risotto that usually turns out so dismal a failure in the ordinary stewpot. A great advantage of the Welbank is that it can be left for hours without attention, and that it is economical so far as gas is concerned. To the country practitioner, to the medical student, to anyone, in fact, who wishes an apparatus in which a hot meal can be easily and efficiently prepared, we can cordially commend the Welbank boilerette. It is cheap, economical, safe, and at a pinch it may even be made to serve as an up-to-date steam steriliser!

## Figures and Tables

**Figure f1:**